# Factors influencing medication adherence among hypertensive patients in primary care settings in Central Vietnam: A cross-sectional study

**DOI:** 10.1371/journal.pone.0307588

**Published:** 2025-01-28

**Authors:** Ho Anh Hien, Nguyen Minh Tam, Huynh Van Minh, Tran Binh Thang, Le Phuoc Hoang, Stefan Heytens, Dirk Devroey, Hoang Anh Tien

**Affiliations:** 1 Department of Family Medicine, University of Medicine and Pharmacy, Hue University, Hue, Vietnam; 2 Faculty of Medicine and Pharmacy, Department of Family Medicine and Chronic Care, Vrije Universiteit Brussel, Brussels, Belgium; 3 Department of Internal Medicine, University of Medicine and Pharmacy, Hue University, Hue, Vietnam; 4 Faculty of Public Health, University of Medicine and Pharmacy, Hue University, Hue, Vietnam; 5 Faculty of Medicine and Health Sciences, Department of Public Health and Primary Care, Ghent University, Ghent, Belgium; Green Pasture Hospital, NEPAL

## Abstract

**Background:**

Medication adherence plays a crucial role in effectively managing hypertension, a significant public health concern, especially in regions like Central Vietnam. This study aimed to assess medication adherence levels among hypertensive patients in primary care settings and explore the factors influencing adherence within this specific population.

**Methods:**

We conducted a cross-sectional study to evaluate medication adherence and its determinants among individuals with hypertension in Central Vietnam. Medication adherence was assessed using the 5-item version of the Medication Adherence Report Scale self-report. We collected data on the demographics, medical history, lifestyle, hypertension knowledge, along with the patient beliefs and perceptions about hypertension. Logistic regression analysis was employed to identify the key factors associated with their medication adherence.

**Results:**

Our study revealed that only half of the hypertensive patients adhered to their prescribed medication regimens. Several factors significantly influenced their medication adherence, including age, ethnicity, educational level, home blood pressure monitoring, healthy diet, time since hypertension diagnosis, hypertension knowledge, and patient beliefs. According to the logistic regression analysis, a healthy diet and patient beliefs emerged as primary predictors of medication adherence. Patients who strongly believed in the necessity of medication demonstrated better adherence, while concerns about overuse and harm were linked to lower adherence levels.

**Conclusions:**

This study highlighted the suboptimal levels of medication adherence among hypertensive patients in primary care settings in Central Vietnam. It underscored the urgent need for tailored interventions to address this issue. For the sake of better medication adherence, healthcare providers were suggested to prioritize patient education, address patient beliefs and concerns about medication, and promote the practice of home blood pressure monitoring.

## Introduction

Hypertension remains a significant public health concern in Vietnam, with its prevalence steadily rising over the past decade. Between 2008 and 2019, the incidence of hypertension increased from 25.1% to 33.8%; notably, Central Vietnam showed a higher prevalence, reaching 44.8% among patients aged 40 and above [1–3]. However, the hypertension control rates remain unacceptably low. In 2008, only 10.7% of all hypertensive patients had their blood pressure under control, with a slightly higher rate of 36.3% among those receiving treatment. By 2015, the proportion of controlled hypertension had only slightly increased to 12.2% overall and 36.8% among those undergoing treatment [1,4].

Optimal adherence to antihypertensive medication is crucial for effective hypertension management and reducing the risk of cardiovascular diseases (CVDs) [5,6]. Despite this importance, the proportion of medication adherence (MA) in hypertensive patients in primary care in Vietnam remained consistently low, ranging from 49% to 66% [7,8]. The existing literature highlighted a great number of factors that could potentially influence MA, particularly in chronic diseases [9–12], as outlined by the World Health Organization (WHO) across five dimensions of adherence [9]. Firstly, the health system-related factors encompass aspects such as the adequacy of health insurance coverage, medication affordability, and the quality of healthcare provider-patient communication [9]. Secondly, the patient-related factors include age, gender, socioeconomic status (including occupation and education level), knowledge and understanding of hypertension, beliefs and perceptions about illnesses and medications, as well as cultural and social norms [9]. When patients possess a good understanding of their medical condition, the purpose of their medications, and the potential benefits of treatment, they are more likely to adhere to their prescribed regimen [11]. This understanding is closely tied to patients’ beliefs in the effectiveness and necessity of their medications. When patients trust that their medications will improve their health and well-being, they are more inclined to adhere to the treatment plan. Thirdly, the condition-related factors involve the severity and chronicity of the medical condition, symptom burden, and the complexity of the treatment regimen. Patients with more severe medical conditions or symptoms may perceive a greater need for MA to manage their symptoms, prevent complications, or improve their overall health outcomes. Fourthly, the treatment-related factors consist of the complexity, convenience, and cost of treatment, especially out-of-pocket expenses [9]. Lastly, the social and environmental factors comprise aspects such as ethnicity, family support, living conditions, and lifestyle choices [8–12]. Nonadherence to medication can be the result of a complex interplay of intentional and unintentional causes. Unintentional nonadherence occurs when patients want to adhere but face barriers like limited capabilities or resources, while intentional nonadherence means patients consciously choosing not to follow the treatment recommendations [13]. Understanding these factors in hypertensive patients is vital for developing effective strategies to support MA and improve the patients’ health outcomes [13–15].

However significant the issue is, Vietnam still lacks studies examining MA and its contributing factors among hypertensive patients in primary care settings. Only one relevant study has been conducted in a rural mountainous area within one province in northern Vietnam, and no studies from Central Vietnam have been published.

The findings of this study revealed a relatively low MA rate among hypertensive patients, with no significant differences in MA observed between the two CVD risk groups. The proportion of MA was found to be higher among females and older individuals [7]. However, a lot of crucial information was reported to be still lacking for full comprehension of these issues and effective solutions to reduce the CVD burden in Vietnam. Therefore, we conducted this study with two main objectives: firstly, to evaluate the MA levels among hypertensive patients at community health centers (CHCs) in Central Vietnam, and secondly, to explore the association between the MA levels and various factors influencing the adherence in this patient population.

## Materials and methods

### Study design and population

We conducted a cross-sectional study in Central Vietnam to assess MA and its associated factors among individuals with hypertension. Data collection took place at 16 CHCs across three provinces, each representing a different region in Central Vietnam. These locations were purposely chosen to ensure the representativeness of where hypertensive patients were treated in primary care settings across Central Vietnam in 2023. The first province that we selected was Thua Thien-Hue, which is situated in the north-central coastal area, covers 494,700 square kilometers, has a population of 1,160,200 and a population density of 235 individuals per square kilometer. The second one was Khanh Hoa Province, which represents the south-central coastal area, spans 520,000 square kilometers, and has a population of 1,254,000 and a population density of 241. Thirdly, Lam Dong Province, selected to represent the Central Highlands, covers 978,100 square kilometers, with a population of 1,332,500 and a population density of 136 [16].

In each province, we randomly selected one urban and one rural district to guarantee the regional diversity. Within these districts, we randomly chose two to four CHCs with the purpose of ensuring an adequate number of participants. Potential participants, ranging from 50 to 70 individuals, were randomly identified at intervals of 5 from lists of hypertensive patients at these CHCs, adhering to specific inclusion criteria. Eligible participants were individuals aged 40 to 75, diagnosed with primary hypertension and receiving treatment at the study site for a minimum of six months. Exclusion criteria comprised secondary hypertension, pregnancy-related hypertension, acute symptoms, and intellectual or cognitive impairments, as had been defined in the study protocols.

### Sample size calculation

To determine the required sample size for our study, we employed a sample size calculation method based on proportions. We referenced a prior Vietnamese study where 49.8% of patients were found to have adherence to the medications [7]. We conducted our sample size calculation with a 5% margin of error and a study power of 99%. Based on these parameters, it was determined that a sample of 660 eligible subjects would be necessary. To make up for the potential non responses, we invited 792 patients to participate in this study. The final sample size in our data analysis was 761, corresponding to the participation rate at 96.1%.

### Data collection

The selected participants were invited to visit their local CHCs during the morning hours at the weekend provided in the invitation letters. Each invitation letter detailed the time, location, and any dietary requirements for the visit. Participants were kindly requested to refrain from consuming food or beverages before their visit for the sake of accurate information regarding their blood pressure (BP).

Data collection was facilitated through a semi-structured questionnaire. Initially, participants were questioned about their demographics and personal behaviors. Subsequently, participants were asked about their knowledge of hypertension, perceptions of the condition, beliefs regarding medication, and adherence to prescribed medications. In the second phase of data collection, anthropometric measurements, including BP and blood cholesterol levels, were recorded. Notably, ten participants had to be excluded from the study due to missing essential information.

Our survey teams comprised medical professionals and students from University of Medicine and Pharmacy, Hue University, in collaboration with local primary healthcare staff. All study investigators and team members had undergone comprehensive training, which familiarized them with the study’s objectives and equipped them with the necessary tools and methods. The survey was carried out over the course of four months, from March 20 to June 30 in 2023.

### Study variables

We collected a range of demographic data, personal and family medical histories related to hypertension, as well as the patients’ history of strokes, coronary heart diseases, and diabetes. We also inquired about the time when hypertension was first diagnosed, and the distance from the participants’ houses to their local CHCs.

For the participants’ physical measurements, we used a digital scale to record their body weight to the nearest 0.1 kg and the Telescopic Measuring Rod to measure their height to the nearest 0.1 cm while they were in a standing position. Their BP was meticulously measured on three occasions, with a minimum interval of 3 minutes, while participants were resting in a seated position. This was done using automatic sphygmomanometers (OMRON HEM 9210T, Omron Healthcare, Tokyo, Japan), equipped with appropriately sized cuffs and following a standard measurement protocol. The analysis relied on calculating the average of the last two measurements [17,18]. To ensure the accuracy of blood cholesterol collection, we followed a carefully designed process. Experienced nurses with at least 2 years of expertise were hired to conduct the blood collection technique, and we utilized qualified laboratories in each province for sample analysis. Patients were instructed to fast for 9–12 hours before the test to obtain precise cholesterol level measurements. Essential supplies such as alcohol swabs, tourniquets, needles, collection tubes, and bandages were gathered for the blood collection procedures. Following standardized guidance, trained nurses collected the blood samples. After the blood collection, we ensured proper storage and transportation of the samples to the laboratories for analysis [18,19]. The results of the blood cholesterol levels were then transferred to us for further evaluation and interpretation.

#### Medication adherence

The primary focus of this study was to assess the patients’ MA, which was evaluated using the 5-item version of the Medication Adherence Report Scale (MARS-5) [13]. The MARS-5 comprises five general statements regarding nonadherent behavior. Participants responded to these statements on a 5-point Likert scale, where 1 denoted "always," 2 represented "often," 3 indicated "sometimes," 4 reflected "rarely," and 5 signified "never." The study’s primary outcome variable was derived from the total score on the MARS-5. The median MARS-5 score was 24. We conducted sensitivity analyses using MARS scores of 23 and 24 as cut-off points to distinguish between non-adherence and adherence. For this study, we specifically chose a cut-off point of 23 because of its high sensitivity (100%) and specificity (100%). Scores ranging from 5 to 23 indicated non-adherence, while scores of 24 to 25 were classified as adherent. The MARS-5 has demonstrated its utility in various studies across multiple countries, consistently exhibiting high validity and reliability [20–23]. For this study, the Cronbach’s alpha coefficient for the MARS-5 (total score) was calculated to be 0.87.

#### Hypertension knowledge

To gauge the patients’ knowledge of hypertension, we employed the Hypertension Knowledge Level Scale (HK-LS) [24]. This instrument comprises 22 items distributed across 6 sub-dimensions, 9 of which contained incorrect statements (items 4, 6, 7, 8, 9, 10, 11, 14, and 17). Participants were awarded one point for each correct answer, while incorrect statements were scored inversely to the other items. Higher scores indicated a greater level of hypertension knowledge. This tool has demonstrated high validity and reliability in previous studies [25,26]. In our study, the Cronbach’s alpha coefficient for the HK-LS (total score) was calculated to be 0.83.

#### Hypertension perception

The patients’ perceptions of their hypertension were evaluated using the Brief Illness Perception Questionnaire (B-IPQ) [27,28]. The B-IPQ comprises 8 distinct items: Consequences, Timeline, Personal control, Treatment control, Identity, Illness concern, Understanding, and Emotional response. Each item was scored on a scale of 0 to 10. To calculate the overall score, items 3, 4, and 7 were reversely coded. A higher total score indicated a more negative perception of hypertension [15,28]. In our study, the Cronbach’s alpha coefficient for the B-IPQ (total score) was calculated to be 0.87.

#### Beliefs in hypertension medicines

The patients’ beliefs regarding their hypertension medications were assessed using the Beliefs about Medicines Questionnaire (BMQ) [27,28]. The BMQ comprises 18 items, divided into 2 parts: BMQ-Specific, which evaluates patients’ beliefs about medication prescribed for their personal use, and BMQ-General, which assesses beliefs about medication in general. The BMQ-Specific consists of two subscales, Necessity and Concern, each containing 5 questions. The BMQ-General has 2 subscales, Overuse and Harm, each with 4 questions. Responses to all 22 questions were recorded on a 5-point Likert scale (ranging from 1 = strongly disagree to 5 = strongly agree), and the total scores were computed for each BMQ scale [27–29]. In this study, the Cronbach’s alpha coefficients for the BMQ-Specific (Necessity and Concern) scales were 0.85 and 0.77, and for the BMQ-General (Overuse and Harm) scales were 0.63 and 0.68, respectively.

#### Translation of questionnaires

The translation process for the MARS-5 and HK-LS questionnaires adhered to the five-stage guideline established by Beaton [28,30]. Two bilingual individuals and two medical doctors conducted forward and backward translation of the questionnaires. They independently translated the original English measures into Vietnamese and subsequently merged these translations into a unified Vietnamese version. Following this, two additional bilingual translators performed a backward translation, converting the Vietnamese version back into English for validation. To assess the clarity and comprehensiveness of the final Vietnamese translations, a pilot trial was conducted involving 30 candidates. The results from this pilot study demonstrated that both questionnaires were clear and easily understood by the participants. Regarding the B-IPQ and BMQ, we also conducted translations specifically for hypertension into Vietnamese. Additionally, we used the Vietnamese versions of IPQ and the BMQ by Thang Nguyen [28] as reference points during the translation process.

#### Other variables

The participants’ occupations were grouped as manual workers (including farmers, traders, housekeepers, and production roles), government staff members, and other occupations (retired or unemployed). The majority were of the ’Kinh’ ethnicity. Urban or rural residence was decided based on administrative boundaries. Education levels were classified as low (primary school or under), intermediate (secondary school), and high (high school or college). Current smokers referred to those using tobacco or having quit smoking within 12 months [18]. Excessive alcohol consumption was defined as consuming more than 2 standard units per day for men or more than 1 standard unit per day for women, with one standard unit typically containing 10 grams of pure alcohol [18]. Adequate fruit and vegetable consumption, reflecting a healthy diet, was defined as consuming at least 5 servings (equivalent to 400 grams) per day, excluding starchy vegetables [18]. Physical activity levels were categorized using metabolic equivalents (METs) per minute per week, where METs represented the ratio of energy expended during an activity to that at rest. Physical inactivity was defined as less than 600 METs per minute per week (MET/min/week) [18]. Assessment of a salty diet relied on the patients’ self-perception, determined through the question, "Do you believe that you consume a higher amount of salty food compared to others in your community?". Body Mass Index (BMI) categories were underweight (<18.5 kg/m²), normal weight (18.5–22.9 kg/m²), overweight (23–24.9 kg/m²), and obesity (≥25 kg/m²) [31]. High cholesterol was defined as total cholesterol ≥ 200 mg/dl [18].

### Statistical analyses

We conducted statistical analyses to evaluate the MA and associated risk factors in hypertensive patients at CHCs in Central Vietnam. All analyses were two-sided, with statistical significance set at p < 0.05. Continuous variables, including Time since hypertension diagnosis, Distance from the patient’s house to their local CHC, Hypertension knowledge, BMQ-Specific (Necessity and Concern), BMQ-General (Overuse and Harm), B-IPQ and each subscale of B-IPQ, MARS-5 and each subscale, were compared using the t-test or Mann-Whitney U test if the data met ordinal assumptions. Categorical variables, including Age group, Gender, Residence, Ethnicity, Living arrangement, Educational level, Occupation, Body mass index, History of CVDs, Home BP monitoring, Number of antihypertensive medication, Number of classes of antihypertensive medication, Health insurance, Patient’s lifestyle including Smoking, Salty diet, Healthy diet, Excessive alcohol use, Physical inactivity, and Hypertension control, were assessed with the Chi-square tests. We identified variables statistically significantly associated with MA with p < 0.05. Subsequently, we used these variables to perform multivariate logistic regression analysis to control for several factors simultaneously and explore adherence predictors. For categorical covariates, Age group (< 60 years), Ethnicity (Majority), Living arrangement (Living with family), Health insurance (No), Healthy diet (No), Physical inactivity (No), Home BP monitoring (No), BP monitoring equipment (No), Educational level (Low level), and Occupation (Manual workers) were chosen as reference categories. After checking the linearity assumption, Time since hypertension diagnosis, Hypertension knowledge score, and the BMQ subscale scores were included as continuous covariates in the logistic regression models. Results were presented as odds ratio (OR) with 95% confidence interval (CI).

During our data collection process, we used the EpiData entry software (version 3.1, EpiData Association, Denmark) for accuracy. The STATA MP software (version 17, StataCorp LLC, Texas, USA) was used for descriptive, analytical, and multivariate logistic regression analyses. Cronbach’s α was employed to assess the internal consistency within the BMQ-Specific, MARS-5, and HK-LS, B-IPQ with α > 0.7 indicating strong internal consistency within subscales or scales.

### Ethical approval

The ethical approval for this research endeavor was granted by the University of Medicine and Pharmacy, Hue University, under the reference number H2023/027. Additionally, documented approvals were obtained from the health departments of three provinces, specifically Thua Thien-Hue, Khanh Hoa, and Lam Dong, with reference numbers 3033/SYT-NVY, 1228/SYT-NVYD, and 1089/SYT-NVY, respectively. The study’s objectives and procedures were comprehensively explained to all prospective participants, and their written informed consent was obtained prior to their engagement in the study. It was crucial to emphasize that all participants retained the unequivocal right to withdraw from the study at any point in time, ensuring the protection of their autonomy and rights throughout the research process.

## Results

### General characteristics of participants

The study involved 761 participants, comprising 55.7% males, with 68.2% aged 60 years or older. Approximately 52.4% of the participants resided in rural areas, and a significant majority (92.5%) identified as ethnically Kinh. Nearly all participants lived with their families, and only 7.1% reported to be living alone. The educational levels were generally low to intermediate, with more than half receiving less than 9 years of schooling. Occupationally, nearly 70% of the participants engaged in manual labor, while one-quarter were either retired or unemployed. Notably, 21.9% and 22.1% of the participants were classified as overweight and obese, respectively (Table 1).

**Table 1:** Demographic and clinical characteristics of patients stratified by medication adherence levels.

Characteristics	Total n (%)	Non-adherence n (%)	Adherencen (%)	p-value
**All patients**	**761 (100)**	**378 (49.7)**	**383 (50.3)**	
***Age group***				0.046
*40–59*	242 (31.8)	133 (55.0)	109 (45.0)	
*≥60*	519 (68.2)	245 (47.2)	274 (52.8)	
***Gender***				0.17
*Men*	337 (44.3)	158 (46.9)	179 (53.1)	
*Women*	424 (55.7)	220 (51.9)	204 (48.1)	
***Residence***				0.08
*Urban*	362 (47.6)	168 (46.4)	194 (53.6)	
*Rural*	399 (52.4)	210 (52.6)	189 (47.4)	
***Ethnicity***				0.001
*Ethnic majority*	704 (92.5)	338 (48.0)	366 (52.0)	
*Ethnic minorities*	57 (7.5)	40 (70.2)	17 (29.8)	
***Living arrangement***				0.013
*Living alone*	54 (7.1)	18 (33.3)	36 (66.7)	
*Living with family*	707 (92.9)	360 (50.9)	347 (49.1)	
***Educational level***				<0.001
*Low*	240 (31.5)	142 (59.2)	98 (40.8)	
*Intermediate*	227 (29.8)	94 (41.4)	133 (58.6)	
*High*	294 (38.7)	142 (48.3)	152 (51.7)	
***Occupation***				0.72
*Manual workers*	510 (67.0)	250 (49.0)	260 (51.0)	
*Government staff*	47 (6.2)	22 (46.8)	25 (53.2)	
*Retired/unemployed*	204 (26.8)	106 (52.0)	98 (48.0)	
***Body mass index***				0.42
*Underweight*	33 (4.3)	192 (48.9)	201 (51.1)	
*Normal weight*	393 (51.6)	21 (63.6)	12 (36.4)	
*Overweight*	167 (21.9)	84 (50.3)	83 (49.7)	
*Obesity*	168 (22.1)	81 (48.2)	87 (51.8)	
***History of stroke***				0.24
*Yes*	57 (7.5)	28 (49.1)	29 (50.9)	
*No*	680 (89.4)	334 (49.1)	346 (50.9)	
*Unknown*	24 (3.2)	16 (66.7)	8 (33.3)	
***History of coronary heart diseases***				0.12
*Yes*	64 (8.4)	27 (42.2)	37 (57.8)	
*No*	540 (71.0)	263 (48.7)	277 (51.3)	
*Unknown*	157 (20.6)	88 (56.1)	69 (43.9)	
***History of diabetes***				0.31
*Yes*	110 (14.5)	58 (52.7)	52 (47.3)	
*No*	544 (71.5)	261 (48.0)	283 (52.0)	
*Unknown*	107 (14.1)	59 (55.1)	48 (44.9)	
***Family history of cardiovascular diseases***				0.033
*Yes*	336 (44.2)	87 (45.5)	104 (54.5)	
*No*	365 (48.0)	179 (48.8)	188 (51.2)	
*Unknown*	60 (7.9)	112 (55.2)	91 (44.8)	
***Home blood pressure monitoring***				<0.001
*Yes*	351 (46.1)	148 (42.2)	203 (57.8)	
*No*	410 (53.9)	230 (56.1)	180 (43.9)	
***Blood pressure monitoring equipment***				<0.001
*Yes*	329 (43.2)	138 (41.9)	191 (58.1)	
*No*	432 (56.8)	240 (55.6)	192 (44.4)	
***Number of antihypertensive medication***				0.37
*1*	607 (79.8)	309 (50.9)	298 (49.1)	
*2*	139 (18.3)	63 (45.3)	76 (54.7)	
*3*	15 (2.0)	6 (40.0)	9 (60.0)	
***Number of classes of antihypertensive medication***				0.43
*1*	661 (86.9)	334 (50.5)	327 (49.5)	
*2*	66 (8.7)	27 (40.9)	39 (59.1)	
*3*	10 (1.3)	4 (40.0)	6 (60.0)	
*Unknown*	24 (3.2)	13 (54.2)	11 (45.8)	
***Health insurance***				0.034
*Yes*	732 (96.2)	358 (48.9)	374 (51.1)	
*No*	29 (3.8)	20 (69.0)	9 (31.0)	
***Smoking***				0.54
*Yes*	176 (23.1)	91 (51.7)	85 (48.3)	
*No*	585 (76.9)	287 (49.1)	298 (50.9)	
***Salty diet***				0.097
*Yes*	207 (27.2)	113 (54.6)	94 (45.4)	
*No*	554 (72.8)	265 (47.8)	289 (52.2)	
***Healthy diet***				0.002
*Yes*	479 (62.9)	217 (45.3)	262 (54.7)	
*No*	282 (37.1)	161 (57.1)	121 (42.9)	
***Excessive alcohol use***				0.85
*Yes*	21 (2.8)	10 (47.6)	11 (52.4)	
*No*	740 (97.2)	368 (49.7)	372 (50.3)	
***Physical inactivity***				0.007
*Yes*	467 (61.4)	214 (45.8)	253 (54.2)	
*No*	294 (38.6)	164 (55.8)	130 (44.2)	
***High total cholesterol***				0.713
*Yes*	222 (58.7)	230 (60.1)	452 (59.4)	
*No*	156 (41.3)	153 (39.9)	309 (40.6)	
***Hypertension control***				0.105
*Yes*	423 (55.6)	199 (52.5)	224 (59.5)	
*No*	338 (44.4)	179 (47.5)	159 (41.5)	
***Distance from home to community health centers*** *(mean(km)± SD)*	2.2 *±* 1.5	2.3 *±* 1.6	2.2 *±* 1.4	0.36
***Time of diagnosis*** *(mean (year) ± SD)*	5.8 *±* 5.1	5.3 *±* 5.0	6.4 *±* 5.1	0.004
***Hypertension knowledge*** *(mean± SD)*	14.9 *±* 4.6	13.8 *±* 5.3	15.9 *±* 3.5	<0.001

SD: Standard Deviation.

The majority of the hypertensive patients in our study (86.9%) took one class of antihypertensive medication. A smaller proportion (8.7%) used two classes of antihypertensive medication, either as two separate pills or combined into one pill. Meanwhile, 18.3% of the hypertensive patients took two pills, but both contained the same class of antihypertensive medication. Almost half of the participants had BP monitoring equipment and regularly monitored their BP at home (Table 1).

### Medication adherence among hypertensive patients in primary care

Half of the hypertensive patients adhered to their prescribed medications, with variations in adherence among demographic groups. The Kinh ethnic majority had a higher rate (52%) compared to ethnic minorities (29.8%). Older patients (52.8%) adhered more than younger ones (45%). Living alone (66.7%) showed higher adherence than living with family (49.1%, p < 0.001). Patients with BP monitoring devices (59.1%) and those monitoring BP at home (57.8%) had better adherence (p < 0.001).

Gender, residence, occupation, BMI, and medical history of CVDs had little impact on the investigated patients’ MA. The number of antihypertensive medications and the number of classes of antihypertensive medications, smoking, salty diet, and excessive alcohol consumption did not significantly affect their adherence, either. However, consuming 5 servings of fruits and vegetables (54.7%) and physical inactivity (54.2%) were associated with higher adherence. Finally, the patients’ MA correlated with hypertension duration and knowledge (Table 1).

### The influences of patient beliefs and perceptions on medication adherence

Table 2 presents findings on the patient beliefs regarding medication necessity and adherence. The patients highly valued medication necessity, scoring an average of 18.3 (SD 3.8). Those with higher scores on the BMQ-Specific subscale for medication necessity exhibited significantly better adherence (mean 19.5, SD 3.5) than those with lower scores (mean 17.2, SD 3.8, p < 0.001). Contrarily, lower scores on medication concerns were associated with higher adherence (mean 13.5 and SD 3.8 versus mean 15.3 and SD 3.7, p = 0.006). The high scores on the General Beliefs subscale, specifically in general overuse and general harm, correlated with reduced adherence (mean 11.6, SD 2.4, and mean 10.4, SD 2.9, respectively), compared to lower scores (mean 10.0, SD 2.6, and mean 9.5, SD 2.7, p < 0.001 for both).

**Table 2:** Patients’ beliefs about medicines and patient’s illness perception by level of adherence among hypertensive patients.

Variables	Total mean(SD)	Non-adherence mean (SD)	Adherence mean (SD)	p-value
***BMQ-Specific***				
*Necessity*	18.3 (3.8)	17.2 (3.8)	19.5 (3.5)	<0.001
*Concern*	14.4 (3.8)	15.3 (3.7)	13.5 (3.8)	0.006
***BMQ-General***				
*Overuse*	10.8 (2.6)	11.6 (2.4)	10.0 (2.6)	<0.001
*Harm*	9.9 (2.8)	10.4 (2.9)	9.5 (2.7)	<0.001
***Brief IPQ***	42.7 (7.7)	41.9 (7.8)	42.6 (7.7)	0.17
*B-IPQ1*. *Consequences*	6.7 (2.7)	6.7 (2.6)	6.7 (2.8)	0.95
*B-IPQ2*. *Timeline*	8.3 (2.3)	7.9 (2.4)	8.7 (2.0)	<0.001
*B-IPQ3*. *Personal control*	6.9 (2.3)	6.6 (2.4)	7.2 (2.3)	<0.001
*B-IPQ4*. *Treatment control*	8.2 (2.0)	8.0 (2.0)	8.4 (1.9)	<0.001
*B-IPQ5*. *Identity*	5.6 (2.6)	5.6 (2.6)	5.7 (2.6)	0.60
*B-IPQ 6*. *Illness concern*	8.3 (2.1)	8.0 (2.2)	8.7 (2.0)	<0.001
*B-IPQ 7*. *Understanding*	7.0 (2.5)	6.5 (2.5)	7.4 (2.3)	<0.001
*B-IPQ 8*. *Emotional response*	5.8 (2.9)	5.8 (2.8)	5.9 (3.0)	0.39

SD: Standard Deviation, BMQ: Beliefs about Medicines Questionnaire, B-IPQ: Brief-Illness Perception Questionnaire.

Patients with higher scores on the "Timeline" dimension demonstrated better adherence (mean 8.7, SD 2.0) than those with lower scores (mean 7.9, SD 2.4, p < 0.001). Additionally, higher scores on "Personal Control" and "Understanding" correlated with improved adherence (mean 7.2, SD 2.3) compared to lower scores (mean 6.6, SD 2.3, p < 0.001). Patients also scored high in "Illness Concern" and "Treatment Control" (mean 8.3, SD 2.1, and mean 8.2, SD 2.0), both correlating with increased adherence. The impact of hypertension was reflected in the "Consequences" score (mean 6.7, SD 2.7), while fewer symptoms (mean 5.6, SD 2.6) and less emotional impact (mean 5.8, SD 2.9) were reported. No significant differences were observed in "Consequences", "Identity", and "Emotional response" scores concerning MA. After reversing scores for personal control, treatment control, and illness understanding, the total illness perception score was 42.7 (SD 7.7) (Table 2).

Table 3 presents the frequency of specific behaviors contributing to medication non-adherence among the hypertensive patients in the present research. The most common behavior associated with medication non-adherence was occasionally ’forgetting to take medicine,’ with a mean score of 3.1 (SD 1.0) on the MARS-5. Following closely were the behaviors of ’interrupting medication intake temporarily’ and ’choosing to skip a dose,’ both with mean scores of 3.7 (SD 1.3). Hypertensive patients reported rare occurrences of ’altering the prescribed dose’ or ’taking less medication than instructed,’ with mean scores of 4.2 (SD 1.2).

**Table 3:** Description of the Medication Adherence Report Scale-5 among hypertensive patients.

MARS-5	Scale score mean			p-value
	Total mean (SD)	Non-adherence mean (SD)	Adherence mean (SD)	
I forget to take medicine	4.0 (1.2)	3.1 (1.0)	4.8 (0.4)	< 0.001
I alter the dose	4.6 (1.0)	4.2 (1.2)	5.0 (0.1)	< 0.001
I stop taking medicine for a while	4.3 (1.1)	3.7 (1.3)	5.0 (0.1)	< 0.001
I decide to miss out a dose	4.3 (1.1)	3.7 (1.3)	5.0 (0.1)	< 0.001
I take less than instructed	4.6 (1.0)	4.2 (1.2)	5.0 (0.1)	< 0.001
Total	21.8 (4.4)	18.8 (4.5)	24.8 (0.4)	< 0.001

**MARS:** Medication Adherence Report Scale, (SD: Standard Deviation.

In our logistic regression analysis, we found a strong connection between the patients’ beliefs about their medication and adherence to treatment plans across 4 domains, as had been assessed by the BMQ-specific subscale. Perceiving medication as necessary had a significant positive association with adherence (OR: 1.26, 95% CI: 1.20–1.33, p < 0.001). Conversely, concerns about medication use were linked to lower adherence (OR: 0.89, 95% CI: 0.85–0.94, p < 0.001). General beliefs about overuse (OR: 0.73, 95% CI: 0.67–0.80, p < 0.001) and potential harm (OR: 1.10, 95% CI: 1.02–1.18, p < 0.001) also influenced MA. Other factors, including family size, adherence to a healthy diet, and regular physical activity, were also significantly associated with MA (Table 4).

**Table 4:** Factors associated with medication adherence—logistic regression analysis results for patients.

Predictor variables	Odds ratio	95% confidence interval (CI)	p-value
**Age group** (≥ 60 years versus < 60 years)	1.14	(0.78–1.66)	0.495
**Ethnicity** (Minority versus Majority)	0.64	(0.32–1.29)	0.212
**Living arrangement** (Living alone versus Living with family)	2.81	(1.38–5.70)	0.004
**Health insurance** (Yes versus No)	1.96	(0.75–5.14)	0.171
**Healthy diet** (Yes versus No)	1.79	(1.25–2.56)	0.002
**Physical inactivity** (Yes versus No)	1.56	(1.10–2.23)	0.013
**Home blood pressure monitoring** (Yes versus No)	1.09	(0.58–2.04)	0.799
**Blood pressure Monitoring equipment** (Yes versus No)	0.74	(0.40–1.39)	0.354
**Educational level** Low level	Rf		
Intermediate level	1.79	(1.14–2.81)	0.012
High level	1.26	(0.81–1.95)	0.297
**Occupation** Manual workers	Rf		
Government staff	0.86	(0.46–1.60)	0.624
Retired/unemployed	0.73	(0.36–1.47)	0.379
**Time of diagnosis** (per 1 year)	1.01	(0.98–1.05)	0.532
**Hypertension knowledge**	0.88	(0.60–1.30)	0.527
**BMQ-specific necessity**	1.26	(1.20–1.33)	<0.001
**BMQ-specific concern**	0.89	(0.85–0.94)	<0.001
**BMQ-general overuse**	0.73	(0.67–0.8)	<0.001
**BMQ-general harm**	1.10	(1.02–1.18)	0.009

BMQ: Beliefs about Medicines Questionnaire, Rf: Reference.

## Discussion

### General characteristic of participants

This research investigated the MA levels among hypertensive patients under the care of the CHCs in Central Vietnam. Furthermore, it sought to examine the associations between MA and various factors influencing adherence within this specific patient population. Our study was the first one to explore this topic in Central Vietnam, in the hope that it would be able to provide comprehensive insights into MA in primary care settings in Vietnam, offering valuable information on this important aspect of healthcare management.

This study involved 761 participants, the majority of which were men (55.7%), and a substantial portion (68.2%) aged 60 years or older. Our findings also highlighted that a significant proportion of elderly hypertensive patients sought care at CHCs, probably owing to the short distance from their houses to the local CHCs (mean distance of 2.2 km), which meant easy accessibility. Additionally, most hypertensive patients in our study did not exhibit symptoms, and over 80% of them were prescribed with only one antihypertensive medication, which could influence their healthcare-seeking behavior. The MA rate among minority ethnic groups was lower compared to the majority ethnicity, aligning with findings from previous studies worldwide [32]. This disparity may be attributed to factors such as lower income, limited education, and restricted access to healthcare services among ethnic minorities, all of which could contribute to lower MA rates [32].

### Medication adherence among hypertensive patients in primary care settings

In our study, only half of the hypertensive patients adhered to prescribed medication. Adherence varied by age groups, with older patients (52.8%) showing higher adherence than young adults (45%); however, no significant gender difference was observed. Our findings aligned with a study in northern Vietnam where the prevalence of MA was reported at 49.8% [7], as well as with results from a meta-analysis of adherence in selected databases (where adherence across all studies was 48% and 57%) [12,33]. Particularly, another study indicated lower adherence levels in Africa (27.6%) and Asia (56.5%) compared to Europe (63.4%) and America (63.4%) [34]. Regional disparities may be attributed to diverse populations, ethnicities, sample sizes, questionnaires, and patient beliefs. However, our findings underscored the significant concern of medication non-adherence among hypertensive patients in primary care settings.

It was observed in our study that older hypertensive patients exhibited higher adherence rates compared to younger ones. This finding aligned with previous studies conducted in Romania and Ghana [35,36]. Older patients typically had more experience managing hypertension, leading to better understanding and adherence to medication regimens. Their years of interaction with healthcare systems and health management were found to contribute to higher health literacy levels among this demographic population. Furthermore, older patients tended to perceive their health conditions, particularly hypertension, as more severe due to potential complications associated with aging. This heightened awareness was reported to motivate them to strictly adhere to prescribed medications for more effective management of their condition. Additionally, older individuals often benefited from stronger family support systems, which included reminders to take medications regularly [32,37]. At the same time, non-adherence to medication regimens among younger hypertensive patients were found to stem from various factors. These included a lack of symptomatic awareness, busy lifestyles characterized by work, family responsibilities, and social engagements, concerns about medication side effects, and limited health literacy [9,32,37]. Therefore, it was imperative to address these obstacles among younger hypertensive adults to effectively reduce their CVD burden in the long term.

Our study also highlighted the importance of home BP monitoring (HBPM) in enhancing hypertensive patients’ MA. HBPM would empower individuals to actively manage their hypertension through key mechanisms. Firstly, it would raise the patients’ awareness through regular at-home measurements, deepening their understanding of their condition and its variations. Secondly, the visual feedback loop from HBPM would motivate the patients as they directly observed how their prescribed medications affect their BP. Our findings aligned with established guidelines recommending HBPM as a valuable tool in hypertension management [17,38,39]. Patients with health insurance reportedly exhibited higher adherence rates, likely thanks to several key factors. Health insurance would provide financial coverage for medical services, including a significant portion of medication costs, thereby enhancing affordability and ensuring consistent access to prescription drugs [32]. Additionally, having health insurance would facilitate the continuity of care, enabling patients to establish long-term relationships with healthcare providers, which proved crucial for ongoing monitoring and management of their health conditions.

We did not find a significant relationship between the number and classes of antihypertensive medications, smoking, or excessive alcohol consumption, and MA in our study. These findings contrasted with a previous study conducted in Ethiopia [40]. The discrepancy in results might stem from differences in study settings, populations, and the definition of variables. The study in Ethiopia was conducted at Jimma University Specialized Hospital, a tertiary care center where hypertensive patients often had multiple comorbidities and required a higher number of antihypertensive medications. It was important to note that adherence to medications was closely linked to adherence to lifestyle modifications, both of which played crucial roles in effective hypertension management. By examining both patient lifestyle behaviors and clinical characteristics, healthcare providers would be able to gain valuable insights to make informed decisions and optimize hypertension management outcomes [17,32].

Our logistic regression analysis identified two patient behavioral predictors of MA: maintaining a healthy diet and physical inactivity. Patients who included fruits and vegetables in their diet might prioritize health-conscious choices, underlining the importance of lifestyle decisions and MA in managing health conditions. Additionally, patients with good habits who actively made dietary changes for health improvement were more likely to engage with healthcare providers and follow medical recommendations, including MA [32]. Interestingly, physically inactive patients also exhibited higher adherence rates, which might be influenced by older patients’ overall greater adherence levels. This finding resonated with the high proportion of physical inactivity (61.4%), similar to the proportion of older individuals (68.2%) in our study. Older patients demonstrated higher adherence compared to younger adults, despite being less physically active. It was important to note that physical activity had proved beneficial for hypertension management and overall health, even in older age groups. Therefore, encouraging suitable exercise among older patients was crucial. The relationship between lifestyle factors and adherence was complex and influenced by various factors such as patient beliefs, perceptions, and communication with healthcare providers. Recognizing the multifaceted nature of this relationship was essential for developing effective strategies to promote MA and improve the overall health outcomes [9,14,37,41–44].

### The influence of patient beliefs and perceptions on medication adherence

A significant link between the patient beliefs and their MA was revealed in our study results. Most patients strongly believed in the necessity of antihypertensive medication, which was evidenced by their mean score of 18.3 (SD 3.8). The hypertensive patients with higher scores on the BMQ-Specific subscale, emphasizing medication necessity, demonstrated better adherence. On the contrary, those with lower scores on the BMQ-Specific subscale, focusing on medication concerns, had higher adherence levels. Similarly, the patients with high scores in the General Beliefs subscale, especially regarding overuse and harm, exhibited reduced adherence, and this finding aligned with previous research [14,29,41,43].

Studies had indicated that perceiving medication as essential for health management led to better adherence. High scores on the BMQ’s "Necessity" subscale, reflecting strong belief in medication importance, correlated with better adherence. Excessive concerns about medications, on the other hand, might lead to skipping doses or discontinuation, acting as barriers to adherence [14,29,41,43]. Factors influencing patient beliefs included doctor-patient communication, health literacy, cultural and social factors, and past medication experiences. Healthcare providers should recognize these beliefs and collaborate with patients to address concerns, provide reassurance, and offer education to improve their MA [14,23,41].

In our study, 5 out of 8 domains in hypertension perception correlated with MA: “Timeline”, “Personal control”,”Treatment control”, “Illness concern”, and “Understanding”. Higher scores in these domains indicated improved adherence, and this proved consistent with prior research [45–48]. A longer perceived timeline signified the patients’ recognition of hypertension as a chronic condition requiring continuous management, enhancing adherence. Patients with a strong sense of personal control tended to proactively manage their health, trusting that their choices, including MA, would positively influence their well-being. Trust in medication and a good understanding of their condition were reported to motivate the patients under research to follow their treatment plans. These insights offered practical implications for healthcare providers. Tailoring interventions to address specific areas of concern related to these domains could effectively promote better MA among hypertensive patients [24,42,45–48].

The most frequently reported behavior for medication non-adherence in our study was "forgetting to take the medicine," aligning with the lowest average score on the MARS-5. The second and third most prevalent causes of non-adherence were "temporarily interrupting medication intake" and "deliberately skipping a dose". This highlighted the importance of interventions that prioritized reminders and the provision of accurate information to support hypertensive patients in adhering to their treatment regimens [49–51].

### Limitations and strengths

Like other cross-sectional studies, our research had several limitations. First of all, using a lengthy questionnaire to assess MA and related factors among hypertensive patients might have led to recall challenges due to its extensive nature; nevertheless, we created a comfortable environment for the questionnaire administration and offered nominal compensation (approximately $3) to acknowledge the participants’ time and effort. Secondly, because this study was conducted at CHCs in Central Vietnam, the findings might not fully generalize to other regions or healthcare settings, given potential differences in infrastructure, access, and patient populations. Thirdly, the data collection tools, specifically the MARS-5, HK-LS, BMQ, and B-IPQ, had not been validated for use in the Vietnamese hypertensive population. To enhance the quality of our study, we meticulously translated these tools following a five-stage guideline and reference documents. We also extensively trained our data collection team, which comprised medical students and local healthcare providers.

At the same time, our study revealed associations but did not determine causality. The salty diet assessment relied on the patients’ self-perception, which might not provide accurate information. Due to the limited resources, we did not investigate the readiness and availability of medicines at the CHCs, nor did we assess the role of healthcare providers in medication management. Further research could be conducted to gather additional information on these aspects.

Despite its limitations, our study was able to provide comprehensive insights into MA and its associated factors among the hypertensive patients in primary care settings in Vietnam. This research presented the first in-depth exploration of this topic in Vietnam, conducted across 16 CHCs in 3 provinces with a large participant pool. The notably low proportion of MA among hypertensive patients, coupled with poor hypertension control rates, highlighted the urgent needs for policymakers, researchers, and primary healthcare providers to promptly address the identified MA issues. Our study proved to be groundbreaking in examining several risk factors influencing MA, including hypertension knowledge, patient perceptions of illness and beliefs about medicine, patient lifestyle factors, and other relevant considerations. Our findings also underscored the critical role of patient knowledge, perceptions, and beliefs in enhancing MA rates. For younger hypertensive patients, targeted interventions should prioritize strategies such as implementing medication reminder systems to improve their MA rates. These insights contributed valuable evidence to the medical literature in Vietnam and emphasized the importance of addressing MA challenges in hypertension management strategies.

### Implications

These findings could serve as valuable guidance for healthcare professionals and policymakers in designing and supporting targeted interventions. Such interventions would encompass the implementation of reminder systems, patient education initiatives emphasizing the importance of MA, and strategies to address concerns and misconceptions regarding hypertension and its treatment. It should be important to emphasize the complex nature of MA, influenced by a multitude of factors, including patient beliefs, perceptions, behaviors, and external circumstances. Consequently, it was recommended that effective interventions adopt a multifaceted approach to comprehensively address these diverse elements.

## Conclusions

MA among hypertensive patients in Central Vietnam remained suboptimal, with approximately only half of the participants classified as adherent. Several factors, including age group, educational level, healthy diet, home BP monitoring, time since hypertension diagnosis, hypertension knowledge, and beliefs about medication, were found to be associated with MA. These findings underscored the importance of patient education, patient-centered care, and tailored interventions to improve MA among hypertensive patients in Central Vietnam. Healthcare providers should prioritize patient education and engage in open and empathetic discussions with patients to address their beliefs and concerns about medication. Future research could explore the availability of medicines at the CHCs and the effectiveness of targeted interventions in improving MA and hypertension management in this population.

## Supporting information

S1 Dataset (XLSX)
